# AN OPTIMIZED MOUSE PARABIOSIS PROTOCOL FOR INVESTIGATION OF AGING AND REJUVENATIVE MECHANISMS

**DOI:** 10.1093/geroni/igac059.1730

**Published:** 2022-12-20

**Authors:** Sonia Rodriguez, Chase Carver, Katayoun Ayasoufi, Felicia Duke Boynton, Marissa Schafer

**Affiliations:** Mayo Clinic, Rochester, Minnesota, United States; Mayo Clinic, Rochester, Minnesota, United States; Mayo Clinic, Rochester, Minnesota, United States; Mayo Clinic, Rochester, Minnesota, United States; Mayo Clinic, Rochester, Minnesota, United States

## Abstract

Surgical parabiosis is widely used to study the mechanistic influence of the circulating milieu on aging and regeneration. This powerful model presents diverse complications based on age, strain, sex, and other experimental parameters. In young (Y) and old (O) male and female C57BL6 mice, we optimized heterochronic (n=12) and isochronic (n=10 Y-Y, n=7 O-O) parabiosis. Throughout protocol development, we identified several complications including variable responses to anesthesia, external and internal dehiscence, dehydration, and weight loss. We identified and implemented solutions during surgical and post-surgical periods, including titrated anesthesia, reinforced internal sutures, topical agents to promote wound healing, and increased supplementation. By consistently adopting protocol changes we were able to significantly increase survival. Separately, we confirmed the time course of chimerism in heterochronic pairs of C57BL6 and Tg(act-EGFP)Y01Osb (eGFP) mice. Baseline and longitudinal blood samples were collected via tail vein. Flow cytometry was used to visualize GFP-positive cells from the parabiont blood sample. Through blood analysis we found that chimerism occurs as early as 2 days post-operatively. Exploitation of our optimized protocol may enable others to efficiently adopt the surgical parabiosis model to dynamically study mechanisms of aging and regeneration.

